# Epistemic injustice, healthcare disparities and the missing pipeline: reflections on the exclusion of disabled scholars from health research

**DOI:** 10.1136/jme-2023-109837

**Published:** 2024-05-23

**Authors:** Joanne Hunt, Charlotte Blease

**Affiliations:** 1 Department of Women's and Children's Health, Uppsala University, Uppsala, Sweden; 2 Digital Psychiatry, Department of Psychiatry, Beth Israel Deaconess Medical Center, Harvard Medical School, Boston, Massachusetts, USA

**Keywords:** Disabled Persons, Ethics, Philosophy, Quality of Health Care, Policy

## Abstract

People with disabilities are subject to multiple forms of health-related and wider social disparities; carefully focused research is required to inform more inclusive, safe and effective healthcare practice and policy. Through lived experience, disabled people are well positioned to identify and persistently pursue problems and opportunities within existing health provisions that may be overlooked by a largely non-disabled research community. Thus, the academy can play an important role in shining a light on the perspectives and insights from within the disability community, and combined with policy decisions, these perspectives and insights have a better opportunity to become integrated into the fabric of public life, within healthcare and beyond. However, despite the potential benefits that could be yielded by greater inclusivity, in this paper we describe barriers within the UK academy confronting disabled people who wish to embark on health research. We do this by drawing on published findings, and via the lived experience of the first author, who has struggled for over 3 years to find an accessible PhD programme as a person with energy limiting conditions who is largely confined to the home in the UK. First, we situate the discussion in the wider perspective of epistemic injustice in health research. Second, we consider evidence of epistemic injustice among disabled researchers, focusing primarily on what philosophers Kidd and Carel (2017, p 184) describe as ‘strategies of exclusion’. Third, we offer recommendations for overcoming these barriers to improve the pipeline of researchers with disabilities in the academy.

## Introduction

 People with disabilities have been described as an ‘unrecognized health disparity population’.^1^ Health disparity (or health inequity) is understood as an avoidable and unjust difference in health or healthcare outcomes experienced by social, geographical or demographic groups with a history of socioeconomic, political or cultural discrimination and exclusion.^1 2^ Despite the passage of landmark disability legislation, including the UK Equality Act 2010, the US Americans with Disabilities Act 1990 and the United Nations Convention on the Rights of Persons with Disabilities (adopted in 2006), disability-related health and healthcare disparities persist. Disabled people report lower levels of well-being on average compared with non-disabled people, are at increased risk of physical and mental comorbidity and are more likely to die younger.^1^,^3^ There are multiple reasons as to why health disparities persist along the lines of disability; however, prejudicial biases, engendering structural barriers to care, play a critical part. For example, recently, the WHO^2^ reported that people with disabilities are significantly more likely to perceive discrimination and stigma in healthcare contexts compared with non-disabled people. This is supported by a wealth of literature from across the world revealing institutional, physical and attitudinal healthcare barriers for disabled people, including medical professionals’ ambivalence or lack of understanding towards disability, lack of confidence vis-à-vis providing quality care and physically inaccessible clinics and clinical equipment.^4^,^7^

Health and healthcare-related disparities also intersect with broader social disparities. For example, people with disabilities are less likely to be employed and earn less when they are in work, despite the fact that disability incurs higher living costs.^2^ In the UK, government data from 2021 reveal a disability employment gap of 28%,^8^ with a disability pay gap of 14%.^9^ Recent figures from the US Bureau of Labor Statistics^10^ indicate that the unemployment rate among disabled people is over twice the rate for non-disabled people, with similar trends across other countries.^2^ Perhaps unsurprisingly, disabled people are also more likely to live in poverty than their non-disabled counterparts.^2 11^ Compounding matters is structural disablism: discrimination and stigma (woven into collective attitudes, organisational policies, legislation and infrastructure) that often go unnoticed by non-disabled people but can take a serious toll on individuals living with disabilities. In 2023, the UK’s Office for National Statistics reported that the suicide rate was higher among people with disabilities than any other demographic group.^12^

To better understand and address such disparities, carefully focused research is needed.^2^ In this regard, people with lived experience of chronic illness and disability can offer unique insights that can strengthen and help drive richer research, where disabled people are positioned equally as co-researchers, as opposed to the traditional dynamic of disabled ‘research subject’ to be passively studied. Through first-hand experience, via experiential or standpoint epistemology,^13^ disabled researchers are often well positioned to understand how health-related policies and practices (informed through largely non-disabled research communities) may unwittingly harm or otherwise disadvantage disabled persons.^14^ Researchers with disabilities may also be more motivated and well placed to perceive knowledge gaps, and to pose penetrating and uncomfortable questions necessary to galvanise change. Embracing viewpoint diversity, and the input of disabled researchers, could therefore represent a powerful pathway to improve understanding and to develop more inclusive health and healthcare policy and practice.

The history of the disabled people’s movement within the UK,^15^,^17^ whereby disabled scholar-activists entered the academy and contributed to profound changes in social practice and policy, constitutes an exemplar of the potential value of viewpoint diversity and disability standpoint, the legacy of which continues today, most notably within disability studies, but also more widely within critical social sciences and humanities.^18^,^20^ However, within health sciences—particularly those tightly tied to science, technology, engineering and mathematics (STEM)—there appear to be greater barriers to including disabled scholars and integrating disabled knowledges.^21^,^23^ For example, research shows that the percentage of people with a declared disability is lower in STEM subjects relative to non-STEM subjects at first degree, postgraduate level and within the academic workforce.^22^ Moreover, a 2020 data analysis brief from the UK All-Party Parliamentary Group on Diversity and Inclusion in STEM^23^ reported that the UK STEM workforce had a lower representation of disabled people relative to the rest of the UK workforce (11% vs 14%). Here, it is noteworthy that the analysis used the wider definition of STEM, that of ‘STEM(H)’ which specifically includes health and related fields.^23^ Such exclusions are further compounded by intersectionality, the intersection and co-constitution of multiple forms of social (dis)advantage.^24^ Indeed, the intersection of disability with other minoritised identities^19 21 23^ is yet another reason to promote disability inclusion within the academy and beyond.

Despite the potential benefits that could be yielded by greater inclusivity, in this paper we describe barriers within the UK academy confronting disabled people who wish to embark on health research. We do this by drawing on published findings, and via the lived experience of the first author (hereafter, ‘JH’) who has struggled for over 3 years to find an accessible PhD programme in the UK as a person with ‘energy limiting conditions’ (ELC)^25 26^ who is largely confined to the home. First, we situate the discussion in the wider perspective of epistemic injustice in health research. Second, we consider evidence of epistemic injustice among disabled researchers, in particular those with ELC, by situating this in the legal context in the UK, and by detailing the nature of barriers experienced. Third, we offer recommendations for overcoming these barriers in the academy.

A note on nomenclature: we recognise that person-first language (‘people with disabilities’) is the globally prevalent form.^18^ As a self-identifying disabled person broadly ascribing to the British social model of disability,^16 17^ JH tends towards identity-first language (‘disabled people’). Therefore, while recognising the semantic and ideological divergences embedded within different forms of disability-related language,^18^ we have chosen to adopt both forms in this paper to reflect our case for viewpoint diversity.

Additionally, while recognising the heterogeneity of disability and disability-related exclusions,^19^ we focus on ELC: health conditions that share energy impairment as a key experience and substrate of disability discrimination or disablism.

ELC include but are not limited to ‘medically unexplained’ or contested conditions such as myalgic encephalomyelitis/chronic fatigue syndrome, alongside ‘rare’ conditions such as Ehlers-Danlos syndromes.^25 26^ Since ELC do not conform to socially prevalent (fixed, non-fluctuating, easily identifiable) stereotypes of disability, disablism largely manifests as clinical and social disbelief, resulting in ELC being poorly recognised and poorly researched through the lens of disability rights and diversity, equity and inclusion (DEI).^25 26^ Equally, while we focus on exclusions within the academic space, it is important to note that people with ELC (and wider disabled communities) are subject to marginalisation and exclusion in all social arenas, including education, employment and the healthcare system itself.^25^,^28^ Moreover, measures to improve physical inclusion (such as wheelchair-accessible environments) are oftentimes ineffective or insufficient among people with ELC who are confined to the home, thus furthering marginalisation of this group. In this respect, we recognise that people diagnosed with mental health conditions (notably but not limited to agoraphobia or social anxiety) may be confined to the home and are subject to similar dynamics of disability-related disbelief and associated exclusions as evidenced in the ELC arena.^29^,^31^ Therefore, while we focus on ELC, the following discussion and recommendations for academic inclusion may benefit others with ‘hidden’ or poorly recognised health conditions.

The importance of ELC-specific research is arguably amplified by the emergence of long COVID, another condition that sits well within the ELC umbrella.^26^ The concept of ELC arose from research led by disabled people within and outside of the UK academy^25 26^ and thus represents an example of the potential value of ‘disability standpoint’ in contributing to health and healthcare-related research gaps. Nevertheless, there is very little peer-reviewed academic literature explicitly focusing on ELC (for recent exceptions see ref 32,34). To our knowledge, and motivating this paper, there is no research exploring academic exclusions in the ELC arena through a lens of epistemic injustice.

## Epistemic injustice

Epistemic injustice refers to a variety of wrongs perpetrated against individuals in their capacity as a knower or contributor to knowledge. According to philosopher Miranda Fricker,^35^ it takes two forms: testimonial injustice and hermeneutic injustice. The former arises when an individual is unfairly discriminated against with respect to their capacity to know or contribute to knowledge. This form of injustice often arises because of negative stereotypes about a demographic group. For example, in the case of disability, testimonial injustice may take the form of global, unjustified prejudices about the intellectual or bodily capacity of disabled individuals to contribute to knowledge. Disabled people may, for example, be seen as lacking the stamina, strength, reliability or acuity to offer useful insights. Philosophers of medicine Ian Kidd and Havi Carel^36^ sum it up as a ‘pre-emptive derogation of the epistemic credibility and capacities of ill persons’ that involves ‘a prior view, for instance, of ill persons being confused, incapable or incompetent, that distorts an evaluation of their actual epistemic performance’. Testimonial injustice can take the form of implicit or explicit discrimination on the part of the hearer, leading to an outright dismissal or discrediting of the contribution of individuals to discussions in which they might otherwise offer valuable insights.

As others have argued, many people with disabilities may have acquired valuable knowledge about their condition through lived experience that renders them experts on aspects of their illness, the nature of health services and the quality of provider care.^27 37 38^ Notwithstanding, it is also important to clarify that living with an illness need not automatically afford epistemic privilege. Rather, the point is that a finer awareness is needed to move past unhelpful stereotyping, to appreciate the contributions to knowledge that individuals may make. This, with a view to avoiding global or unwarranted assumptions about the credibility of individuals’ contributions to knowledge formation activities.

Hermeneutic injustice represents a wrong which Fricker describes as the set of structural and social problems that arise because ‘both speaker and hearer are labouring with the same inadequate tools’.^35^ This form of injustice arises when individuals are precluded from accessing, or can only partially access, resources that could improve understanding about their experiences. Because of this asymmetry, those with unequal access to resources can suffer additional disadvantages that serve to further undermine their status and impede understanding about their condition. Kidd and Carel describe two kinds of means—which they dub ‘strategies’—by which hermeneutic injustice can be explicitly or implicitly perpetuated.^39^ The first includes a range of structural barriers to participation in practices whereby knowledge is formed. Kidd and Carel argue that these can encompass physical barriers and subtler exclusions such as employing specific terminologies and conventions that serve to exclude the participation of disadvantaged people who might otherwise usefully contribute to knowledge.^39^ A related, second strategy of exclusion, they argue, is the downgrading of certain forms of expression (such as first-person experiences, affective styles of presentation or vernacular) as evidence of the diminished credibility of the marginalised group. This demotion, Kidd and Carel contend, serves to further frustrate the efforts of the disadvantaged individual to participate, compounding ‘epistemic disenfranchisement’.^39^ In this way, hermeneutic injustice can lead to a vicious, self-perpetuating cycle of testimonial injustice.

In what follows, we focus primarily on evidence of hermeneutic injustice, including strategies of exclusion among disabled researchers with ELC, who are largely or completely confined to the home and who seek to contribute to knowledge formation activities within the UK academy. Before we delve into the evidence, however, we offer some contextual caveats. First, it is important to offer some legal context with respect to disability rights. On the most charitable analysis, we acknowledge that not every individual who is disabled can expect to participate in every research context. For example, some barriers—such as the design or location of laboratories—might preclude full participation among some disabled researchers even with significant adaptations. Our aim then is to examine forms of epistemic injustice that pertain to ‘reasonable adjustments’, a legal term that we will unpack. Since our focus is on barriers to people with disabilities in British universities, we focus on UK legislation; however, what we have to say doubtlessly applies to other countries and regions.

## Evidence of epistemic injustice among disabled researchers

### Background on UK disability legislation

Under Section 20 of the UK Equality Act 2010, higher education providers in England, Scotland and Wales are legally bound to provide ‘reasonable adjustments’ for people with disabilities who require them.^40^ Section 6 of the Act defines disability as the experience of an impairment that has a ‘substantial’, long-term adverse impact on a person’s ability to engage in daily activities. Section 20 clarifies that the duty to make reasonable adjustments exists where any provisions or criteria offered or required by education providers place disabled people at a ‘substantial’ disadvantage relative to non-disabled people.^40^

Health scholars have identified vagueness and therefore ambiguities in how qualifiers such as ‘substantial’ and ‘reasonable’ are interpreted.^41^ Moreover, it has been contended that ‘reasonable adjustments’ rely on a non-disabled and potentially ableist perspective of what is reasonable, while also placing the burden to prove eligibility for adjustments onto disabled people, thus individualising the structural problem of normalised discrimination.^42^ As previously outlined, ELC are poorly recognised as forms of disability, and research demonstrates that people living with diagnoses that can be positioned as ELC struggle to gain the recognition necessary to obtain reasonable adjustments.^32^,^3443^ Section 19 of the Equality Act 2010 explains that indirect discrimination occurs when one party applies a provision, criterion or practice that puts a person with a protected characteristic (such as disability) at a substantial disadvantage when compared with people without that protected characteristic.^40 44^

The Equality Act allows for scenarios where discrimination may be justified (known as ‘objective justification’) in cases where providers can demonstrate that their policies or provisions constitute ‘a proportionate means of achieving a legitimate aim’.^40^ Among the considerations about what might constitute a proportionate means are the size of the organisation, the practicalities and costs involved.^44^ However, these are seldom explicitly articulated as a justification for the status quo, and the resulting ambiguities (which ultimately can only be resolved by tribunal or court) mean—as we will next find out—that disability discrimination may inadvertently become normalised.

### Evidence of strategies of exclusion

Despite an ostensible increase in DEI policies within the academy,^45 46^ there exists considerable literature demonstrating experiences of physical and attitudinal barriers to participation in academic research among disabled students and academics, including those with diagnoses that sit within the ELC umbrella.^2931^,^34 43 46^ There is also evidence that disability-related inequities in higher education persist in terms of degree completion, degree attainment and progression onto skilled employment or postgraduate study, within and beyond STEM.^21 22 47 48^ The experience of JH is that such disparities are deeply entwined with physical and attitudinal barriers to full epistemic participation within the academy. Drawing on research findings and situating these against the lived experience of JH, we now explore evidence of strategies of exclusion for disabled researchers that, we argue, could contribute to epistemic injustice.

Studies that reveal barriers to academic participation, among people with ELC and disabled people more broadly, focus on two principal scenarios: (1) experiences of higher education students who can attend ‘on campus’ but require accommodations,^29 33 43^ and (2) experiences of academics (from PhD study level upwards) navigating workplace barriers pertaining to reasonable adjustments, employment and career progression opportunities.^31 34 46 49^ Where these barriers occur, we suggest they point to evidence of hermeneutical injustice that may also be underpinned by testimonial injustice. Indeed, chief among themes across such literature is that of ableism, understood as ‘a cultural imaginary and social order centred around the idealised able-bodied and -minded citizen who is self-sufficient, self-governing and autonomous’^50^; this ‘social order’ is founded on global prejudices about disabled bodies and minds.^50^ Reports of academic ableism are evidenced as manifesting through, inter alia, a lack of accessible buildings and equipment, institutional inability or unwillingness to facilitate disability-related accommodations, and lack of familiarity (or consensus) among faculty and non-academic staff as to what constitutes disability-specific DEI practice and policy.^31 43 45 46^ Additionally, increasing literature probes the creeping neoliberalisation of academia, which is contended to intersect with and perpetuate ableism, most notably though institutional normalisation of competition and hyperproductivity as a reflection of ‘excellence’.^31 46^ Relatedly, and notably among students or academics with health conditions that can be positioned as ELC, the question of whether or how to disclose disability and implications of (non)disclosure is receiving critical attention.^21 29 31 33 34 43^

Furthermore, as previously outlined, scarce attention has been paid to ELC explicitly, especially among people with ELC who are largely or completely confined to the home, yet may wish to continue within or enter academic spaces and thus require remote access. JH’s experience is that some of these people are not only marginalised within the academy but may be excluded from accessing it altogether. This, it would appear, is owing to a failure of institutions to facilitate remote access programmes. Here again, to understand how strategies of exclusion operate, we must turn to legal considerations. In terms of what might be considered ‘reasonable’, the willingness of research institutes to extend remote access to students and faculty during successive lockdowns owing to the SARS-CoV-2 pandemic^31 51 52^ suggests that failure to extend such accommodations to disabled people who depend on them, and especially where research can be conducted from home, would be difficult to justify.

Yet, such remote access tends to be considered at best an ‘adjustment’ to preferred or ‘normal’ (non-disabled) practice, and provision appears to be patchy and poorly signposted; lack of clarity over which research institutes offer remote delivery programmes may thus constitute the initial hurdle. Some universities appear to offer remote PhDs within some disciplines but not within others, and the exclusions do not appear to be related to pragmatics such as requiring laboratory access. For example, according to JH’s enquiries, and information received, one UK research institute and member of the Russell Group (representing UK leading research-intensive institutions) offered distance learning PhD programmes in 2021 and 2022 within psychology, but not within sociology. For added context, JH’s research interests are interdisciplinary but primarily straddle disability studies (typically sited within academic schools of sociology and faculties of social sciences) and psychology. This is with a view to researching disability-affirmative, socioculturally and politically cognisant approaches to psychotherapy practice and policy. However, in academic fora, psychology and psychotherapy (often aligned with health sciences faculties) foreground heavily medicalised understandings of disability, and JH’s experience has been that psychology departments have not been open minded or welcoming vis-à-vis the prospect of integrating sociocultural and political perspectives, as per disability studies. In practice, this has meant that JH’s endeavours to find an accessible PhD have been limited to the purview of sociology. These disciplinary exclusions arguably represent the legacy of the reluctance of psychology, wider health sciences and life sciences to embrace disability in all its diversity.^21^,^2350^

In response to an enquiry as to why the above institution did not offer remote access PhDs in disability studies/sociology, the postgraduate admissions team informed JH: ‘All our PhD students undertake mandatory units which are only delivered in person’ (email, 10 February 2022). It is unclear how these mandatory units differ from units offered on remote access programmes. Indeed, a recurring motif throughout JH’s enquiries across various UK institutions is that further probing about potentially exclusionary policies results in ambiguous responses, or no response at all. Reasons for lack of remote access offered by other institutions included a mandatory requirement for direct (on-campus) contact with the PhD supervisor or the need to participate in onboarding sessions face to face on campus. However, lack of justification about why this was necessary was not offered.

Again, it might be expected that institutional willingness to provide remote access during lockdowns would serve as a precedent for remote access to become the norm rather than the exception.^46^ However, in response to JH challenging lack of remote access provision on these grounds, the reply from the admissions team at another Russell Group university was as follows:

While during the last year some teaching and supervision has taken place online this is a temporary measure and not part of a formal distance learning course. Some supervision and teaching is also now taking place back on campus in person again. All ‘on campus’ programmes are subject to government mandated attendance requirements. (email, 28 January 2022)

When JH requested more details regarding these government-mandated attendance requirements, the admissions team declared that the enquiry would be passed onto another point of contact. Over 2 years later, no further details have been forthcoming. Ad hoc adjustments pertaining to remote delivery might be possible at some institutions, but it seems conceivable that these may be dependent on the supervisor’s individual preferences rather than policy, perhaps permitting prejudicial judgements about disability to interfere with decision-making.

Furthermore, for those fortunate enough to find a supervisor willing to ‘accommodate’ them, additional strategies of exclusion arise pertaining to funding via doctoral training programme (DTP) and research council consortiums. For example, a representative of the UK White Rose social sciences DTP^53^ (covering seven UK higher education institutions in Northern England) informed JH that, in accordance with Economic and Social Research Council (ESRC) policy, disabled students confined to the home are not eligible to be considered for funding. Further digging revealed that this policy is not limited to the White Rose DTP; for example, the UK Midlands Graduate School DTP,^54^ covering a further eight UK higher education institutions, lists the same exclusion criteria on its website at time of writing. When JH challenged the White Rose DTP’s policy on grounds of (dis)ableism, a representative forwarded the following response from the ESRC:

UKRI [UK Research and Innovation, non-departmental body of the UK government responsible for funding research] terms and conditions confirm that UKRI funded students must live within a reasonable travel time of their Research Organisation (RO) or collaborative organisation to ensure that they are able to maintain regular contact with their department and their supervisor. This should also ensure that the student receives the full support, mentoring, access to a broad range of training and skill development activities available at their RO, as well as access to the resources and facilities required to complete their research successfully and to a high standard. Our expectation also reflects that we want to avoid students studying in isolation […] (email, 15 December 2022)

In light of the considerable evidence that scholars across many disciplines can work remotely, the assumption that disabled people cannot research to a ‘high standard’ while confined to the home is problematic. Additionally, the reasoning around avoiding isolation, while likely well intended, does not hold much weight from JH’s standpoint. Many disabled people frequently experience significant physical and emotional isolation through navigating a (dis)ableist society and develop numerous strategies (including use of remote access technology) to mitigate this; in this respect, they may even be considered ‘experts by experience’ in resiliently striving to manage isolation.^51 55 56^ Social media, for example, is used by many disabled people to connect with others, share ideas on managing health conditions and disability discrimination and develop collective advocacy and activism initiatives.^55^ Refusing to offer remote access on (partial) grounds that disabled people may not be able to cope with the ensuing isolation risks infantilising people with disabilities, and withholds one of the very tools that can facilitate inclusion and thus counter isolation.

Moreover, literature suggests that being on campus does not necessarily prevent disabled people from experiencing or overcoming isolation, notably emotional isolation or alienation arising from lack of accommodations and thus feeling ‘unwelcome’ or ‘less than’.^33 46^ The ESRC’s reasoning would therefore appear to arise from a non-disabled perspective (or at least, a perspective not attuned to certain facets of disability culture). Funding-related barriers are aggravated by the general lack of other funding opportunities for disabled students. For example, while scholarships for other under-represented groups are justly offered across many institutions,^57^,^59^ often with emphasis on recruiting traditionally marginalised candidates, similar much-needed initiatives for people disadvantaged through disability are conspicuously absent. This is particularly important to address since disability and economic disadvantage are entwined in a complex manner,^2 11^ and because, as previously noted, disability is intersected with other forms of social (dis)advantage.^19 21 24 28^

It is worth emphasising that the exclusionary practices pertaining to health-related research, as discussed here, may be more pervasive and entrenched than we have presented. Discussing the impact of academic ableism, Brown^46^ notes that disability disclosure rates, though on the increase in undergraduate admissions, drop between undergraduate and academic employment level. Brown identifies two factors that might explain this: (a) disabled academics may avoid disclosure for fear that declaring disability would impede their career, and (b) disabled students may simply drop out of the academy. As the foregoing demonstrates, JH’s experience suggests that the second factor may be entwined with disabled students being excluded from the academy because they cannot meet ‘on campus’ attendance requirements. It is currently unknown how many fledgling academics with disabilities have been excluded from the academy owing to discriminatory policies and academic culture, but it seems likely that JH’s case is not exceptional. Recent research recounts that some disabled faculty are being refused remote working arrangements as lockdown accommodations begin to revert to ‘normal’ practice.^60^ For disabled researchers in perpetual lockdown, such refusals might result in experiences such as those detailed here remaining unknown and thus unaddressed.

In summary, where a ‘leaky pipeline’ exists vis-à-vis academic representation of some historically oppressed groups,^61 62^ it appears that there exists no pipeline at all for a subgroup of disabled people who cannot leave their homes due to a combination of body/mind restrictions and lack of social provisions such as healthcare. Yet, disadvantages created by refusing remote access accommodations to scholars with disabilities who are confined to the home are certainly substantial. Beyond the potential loss to collective wisdom, the hermeneutical injustice perpetuated by barriers to education and employment among disabled people results in what Kidd and Carel describe as a ‘double injury’,^39^ since it leads to significant ramifications for the psychosocial well-being and financial security of those excluded.

## Conclusions and recommendations

Despite an ostensible increase in commitment to DEI policy and practice, the academy is far from an inclusive space for disabled people. In the case of disabled people who are unable to leave the home, we might better speak of outright exclusions as opposed to marginalisation. The above discussion has demonstrated that various strategies of exclusion operate within the academy that serve to exclude some people with disabilities ‘from the practices and places where social meanings are made and legitimated’.^39^ Such exclusions risk further marginalising an already hermeneutically marginalised group, with concomitant psychosocial, occupational and financial harms. Additionally, these exclusions incur a loss of collective wisdom that adversely impacts the development of inclusive, safe and effective healthcare practice and policy.

Although we urge the importance of universities facilitating remote access to disabled scholars, we add a note of caution. First, a remote access academy should be offered in complementarity with, as opposed to an alternative to, ensuring accessibility of academic buildings and equipment, or to otherwise supporting disabled people to attend on campus. This is especially important since we also acknowledge that remote access is not a solution for all disabled people.^52 63^ Of note, while remote access can be understood as an assistive technology that helps support the health, well-being and social inclusion of people with disabilities,^2^ the digital divide means that disabled people are also less likely to be able to access this technology compared with their non-disabled counterparts. Such marginalisation is owing to lack of devices, broadband connectivity or reduced digital literacy, underpinned by financial, social and educational disparities as already discussed.^1 2 63^ Our promotion of remote access as an inclusivity tool does not negate the need to address this divide. Nevertheless, recent research has shown that a leading UK online education provider (University of Derby) has three times as many disabled students as the national average,^30^ suggesting that remote delivery of academic programmes can be a significant facilitator of DEI. We therefore conclude by offering recommendations with a view to building on such strategies of inclusion.

Given the lack of familiarity vis-à-vis disability-specific DEI practice and policy, as reported in literature^31 45 46^ and as experienced by JH, our first recommendation is for formalised disability equality training and education initiatives that specifically take account of people with ELC and those confined to the home. Since report of such training reinforcing disability-related stereotyping exists,^31^ there should be greater emphasis on co-producing such resources with people with disabilities, including those confined to the home who are often excluded from public policy-making. Such initiatives, which could also beneficially target personnel involved in research councils and DTPs, should address implicit personal and organisational biases, facilitate understanding of how current policy and practices perpetuate (dis)ableism and promote a proactive approach to equity and inclusion, specifically in the case of people confined to the home. Disabled researchers and disability studies scholars have argued that an institutional culture change is necessary to move beyond a perfunctory engagement in, or basic legal compliance with, DEI initiatives; a foregrounding of the social model of disability and universal design principles has thus been proposed in developing DEI policy and practice.^29 31 46^ The social model upends academically prevalent (individualistic) representations of disability and reasonable adjustments, by placing the onus for change on social structures and institutions as opposed to the people who are discriminated against.^16 17^ In the case of ELC, we suggest that the social structures requiring greatest change to facilitate inclusion are attitudinal contexts, most notably disbelief.^24 25^ In complement to the social model, application of universal design tenets to academic contexts, which involve building ‘accommodations’ into academic standard and managing disability-related diversity proactively as opposed to reactively,^29 31 46^ should be extended to remote access. In practice, this means reducing the likelihood that disabled people have to ask and prove eligibility for reasonable adjustments.^42^

Second, we recommend greater institutional transparency, including clear guidance for researchers with disabilities, vis-à-vis remote working policies. For many research and study programmes, online library access, supervision and other meetings represent acceptable accommodations, if not candidates for integration into academic standard as a complement to on-campus delivery. Such accommodations should be clearly signposted and, where remote working is not possible or government mandates apply, both transparency and strong justifications are required. In this regard, an institution outside of the UK has set a precedent. Uppsala University in Sweden has welcomed JH as research affiliate in the Department of Women’s and Children’s Health, operating entirely via remote access. This approach, which embraces remote working as if it were standard practice (as per universal design principles), is invaluable in challenging the prevalent yet exclusionary academic notion of dominant (on-campus) practice and policy as ‘normal’ and ‘ability neutral’. It thus serves as an exemplar for disability-related best practice for UK institutions.

Third, the current funding system requires considerable revision to better include people with disabilities who are confined to the home. In cases where research projects can be conducted remotely, there is surely no justification for exempting this group of disabled people from being eligible to apply for grants and PhD stipends. As per our above recommendations for remote accommodations, information on funding eligibility should be easily accessible, with strong and transparent rationale for any exclusions. Additionally, existing initiatives to ring-fence funding for researchers from minoritised groups to study health-related inequities^64^ should be extended to include disabled people. Without such measures, much-needed research might never be conducted. This article, which has arisen from disability standpoint, and both disability and academic allyship, has indicated a considerable research gap pertaining to how disabled students or academics confined to the home experience barriers to health-related research. With a view to addressing this research gap with the added value of disability standpoint, funding opportunities must facilitate the inclusion of disabled researchers. Yet, while some under-represented groups are supported through funding-related DEI schemes,^64^ disability is often overlooked.

Finally, we recommend a more formalised and universally applied academic DEI monitoring and ombudsman scheme, both to assess DEI-related shortcomings and to support minoritised researchers in raising concerns. Disabled scholars have suggested using Disability Standard (a form of benchmarking used in business to assess inclusivity and accessibility) to analyse gaps in disability-related DEI practice and policy^31^; practical application across UK universities appears very limited. Existing schemes to promote DEI within the education sector should ensure that disability, including disabled people confined to the home, is represented and consider how institutional compliance can be secured. ‘Advance HE’ is a UK non-governmental body that promotes excellence in higher education, an objective the body acknowledges as entwined with DEI.^65^ While DEI ‘international charters’ pertaining to gender and race exist with a view to encouraging providers to commit to inclusion of under-represented groups,^65^ an equivalent charter specifically for disability does not exist. Here again, we recognise that different forms of discrimination intersect and that race and gender shape disability.^2 21 28^ Moreover, while we do not mean to overlook recent efforts among Advance HE and other bodies to include disability in DEI initiatives,^66^ the voluntary nature of many of these initiatives (which ‘encourage’ higher education institutions to address more fully disability-related DEI) will likely allow the inequitable status quo to persist. Seeking to ground a collective institutional commitment to disability inclusion within legislation, or at the very least within a transparent ‘award’ system as with DEI initiatives pertaining to other under-represented groups,^65^ would likely lend more gravitas to such schemes and ‘nudge’ research institutes towards greater accountability.

In summary, insights from scholars with disabilities can help to inform more inclusive, safe and effective health-related interventions, with further benefits for social inclusion. Current academic structures deny opportunities to the very people who are well placed to identify and research the most overlooked problems in our health systems. If we truly prize DEI, the academy must become more accessible to disabled people.

## Data Availability

Data sharing not applicable as no datasets generated and/or analysed for this study.
